# On-Chip Electrochemical Sensing with an Enhanced Detecting Signal Due to Concentration Polarization-Based Analyte Preconcentration

**DOI:** 10.1021/acs.analchem.4c01018

**Published:** 2024-04-09

**Authors:** Sinwook Park, Daniel Kaufman, Hadar Ben-Yoav, Gilad Yossifon

**Affiliations:** † School of Mechanical Engineering, Tel-Aviv University, Tel Aviv, 6997801, Israel; ‡ Nanobioelectronics Laboratory (NBEL), Department of Biomedical Engineering, Ben-Gurion University of the Negev, Beer-Sheva, 8410501, Israel; § Department of Biomedical Engineering, Tel-Aviv University, Tel Aviv, 6997801, Israel

## Abstract

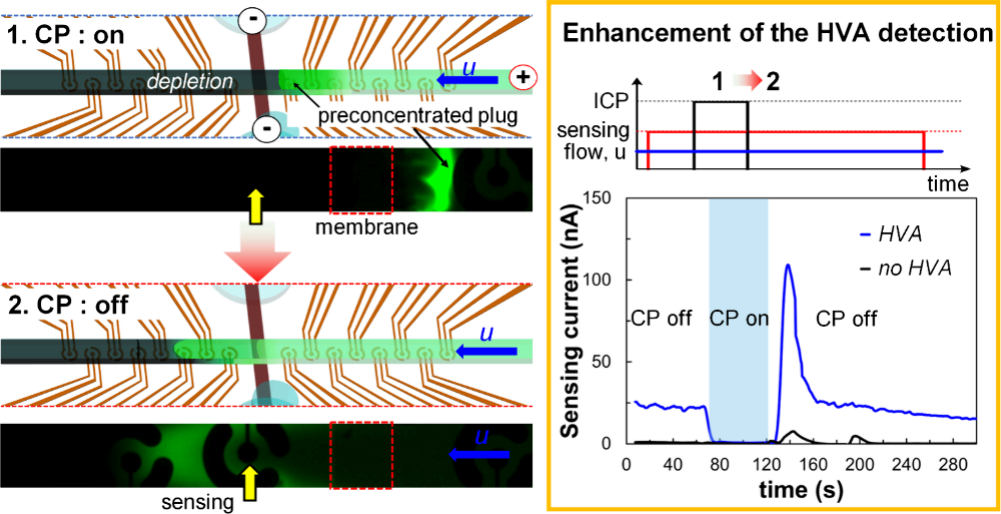

Here, we integrated two key technologies within a microfluidic system, an electrokinetic preconcentration of analytes by ion Concentration Polarization (CP) and local electrochemical sensors to detect the analytes, which can synergistically act to significantly enhance the detection signal. This synergistic combination, offering both decoupled and coupled operation modes for continuous monitoring, was validated by the intensified fluorescent intensities of CP-preconcentrated analytes and the associated enhanced electrochemical response using differential pulse voltammetry and chronoamperometry. The system performance was evaluated by varying the location of the active electrochemical sensor, target analyte concentrations, and electrolyte concentration using fluorescein molecules as the model analyte and Homovanillic acid (HVA) as the target bioanalyte within both phosphate-buffered saline (PBS) and artificial sweat solution. The combination of on-chip electrochemical sensing with CP-based preconcentration renders this generic approach adaptable to various analytes. This advanced system shows remarkable promise for enhancing biosensing detection in practical applications while bridging the gap between fundamental research and practical implementation.

## Introduction

The distinctive ion permselectivity exhibited by ion exchange membranes, primarily attributed to their charged surface groups, plays a pivotal role in selectively permitting the passage of counterions while excluding co-ions. Under nonequilibrium conditions induced by an external electric field, this remarkable phenomenon triggers a symmetry breaking, leading to the creation of ion-depleted and ion-enriched layers at the opposing interfaces between the membrane and the surrounding electrolyte solution, a phenomenon known as ion Concentration Polarization (CP).^1−7^ CP-based preconcentration of analytes, occurring at the edge of the depletion layer due to opposing convective and electromigrative fluxes of the third ionic species, holds immense potential for the advancement of highly sensitive biosensing applications.^8−17^ CP-based preconcentration has proven as be a crucial tool in the development of ultrasensitive biosensors for the rapid detection of disease markers, offering the promise of early disease diagnosis and intervention.^18−24^

Electrochemical sensors shift the systems equilibrium state by applying potential or charge and detecting electron transfer based on reduction–oxidation (redox) properties of the target analyte, thus detecting a specific redox analyte in the solution.^25−27^ These sensors have major advantages such as high sensitivity, speed, and cost-effectiveness.^28−30^ In microfluidic channels, electrochemical sensors can be used and monitored with higher efficiency due to analytes convection, miniaturization, portability, low sample and reagent consumption, and high throughput.^31−33^ Thus, electrochemistry can be a fast and efficient method to detect changes in the analyte concentrations and properties in a flowing system.

The integration of two pivotal technologies within a microfluidic framework, namely CP-based electrokinetic preconcentration of analytes and electrochemical sensors for analyte detection, holds the potential to synergistically amplify detection signals.^34−36^ This is particularly significant for portable point-of-care diagnostic tools, which typically exhibit lower sensitivity compared with centralized laboratories. Recent efforts have explored various strategies for combining electrochemical sensors with CP-based preconcentration.^34,35^ For examples, Hong at al. has developed a microvalve-controlled microfluidic system in which analyte preconcentrated via CP, was isolated using microvalve control and subsequently immobilized on a sensing electrode for electrochemical detection.^34^ Subramanian et al. developed an off-channel CP preconcentrator where multiple ions are preconcentrated at an inlet reservoir and then subjected to signal detection after turning off the CP and applying differential voltammetry.^35^ However, to the best of our knowledge, CP-based electrokinetic preconcentration and electrochemical sensing have been completely decoupled from each other. It is essential to gain a deeper fundamental understanding of their interaction, as potential adverse effects may arise such as cross-talk between simultaneously applied electrically driven CP and electrochemical sensing electric fields as well as gaining continuous monitoring of analytes.

This study presents an on-chip electrochemical sensing system that integrates CP-based preconcentration and local electrochemical sensing, facilitating both decoupled and coupled operation modes for continuous monitoring. We conducted experiments exploring decoupled and coupled configurations through periodic CP operation and electrochemical sensing using chronoamperometry and differential pulse voltammetry (DPV) methods. In a decoupled mode, the electrochemical sensing is turned on only at times when the CP operation is turned off, while in a coupled mode there is a continuous electrochemical monitoring even during the CP operation. These experiments encompassed various sensor locations, target analyte concentrations, and electrolyte concentrations using fluorescein molecules (fluorescence and redox responsive analyte; reductive species is fluorescent, oxidative species is nonfluorescent^37,38^) as a target analyte. Additionally, we enhanced the sensing signal of homocyclic acid (HVA) as a target bioanalyte within both phosphate-buffered saline (PBS) and artificial sweat solution using a coupled configuration. This innovative approach enables continuous monitoring of analytes, offering substantial benefits across a broad spectrum of applications. Combining on-chip electrochemical sensing with CP-based preconcentration of the analyte renders our approach versatile and adaptable to various scenarios, establishing its value in the field of sensor technology.

## Results and Discussion

### Generation of Concentration Polarization (CP)-Based Analyte Preconcentration

**Figure 1:**
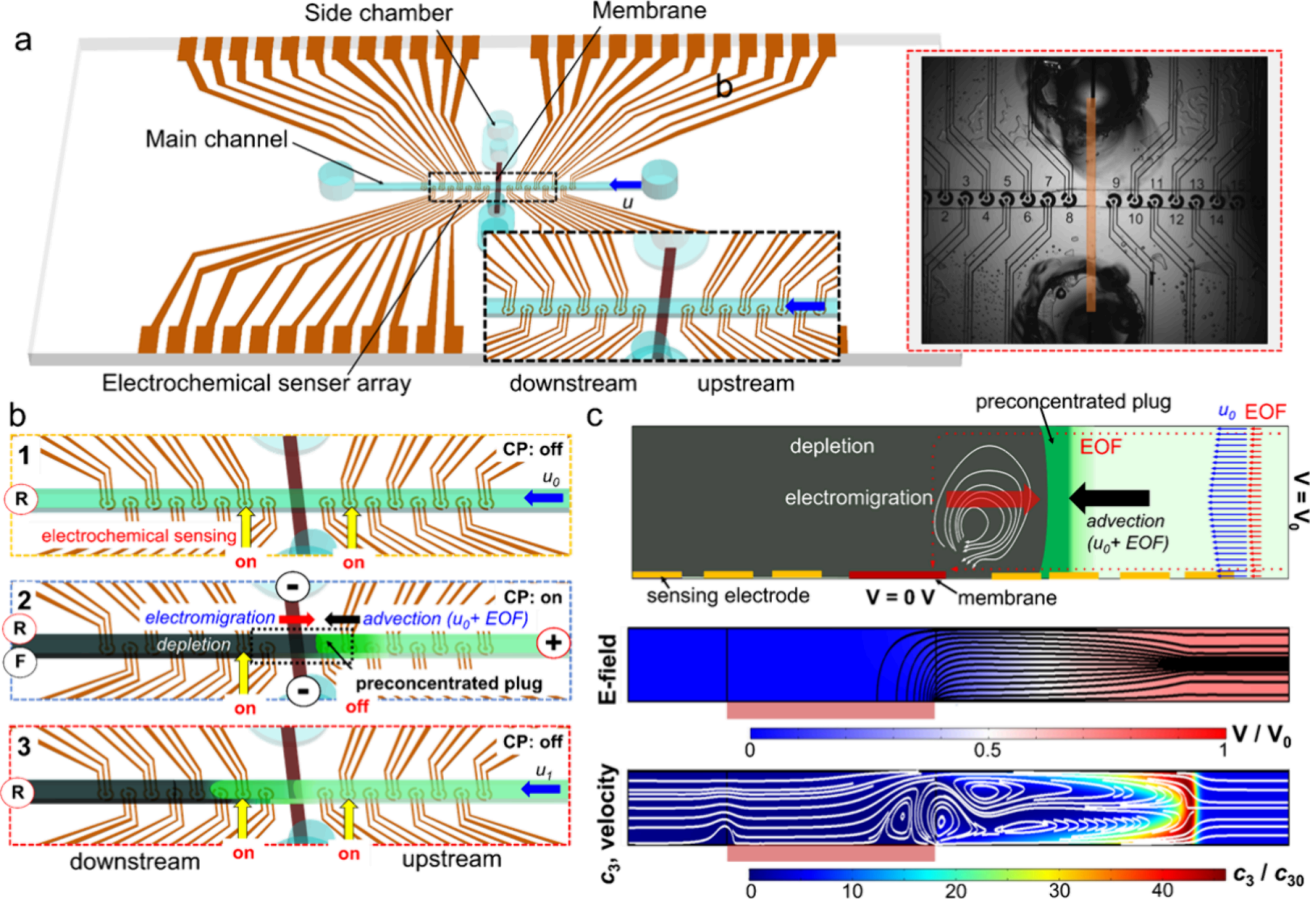
Microfluidic chip combining concentration-polarization (CP) and electrochemical sensing. (a) Schematic of the microfluidic chip with an array of electrochemical sensors with inset of a microscopic image of the fabricated chip. (b) Schematic description showing operational process of both electrochemical sensing and (1) initial state of sensing without CP, (2) I-t sensing location during CP and preconcentration process, and (3) I-t sensing locations after the preconcentrated plug was released downstream. Letters R and F of circle in left end channel indicate the use of external reference electrode (Ag/AgCl) and floating status, respectively. (c) Schematic illustration of CP-based preconcentration of analyte by field-gradient-focusing effect and representative 2D numerical simulation results, indicating electric (black lines) and velocity (white lines) field streamlines along with the third species concentration (*c*_3_). The blue and red arrows in the schematics represent the direction of the pressure-driven (*u*_0_ > 100 μm s^–1^) and induced electroosmotic flow at the upstream channel, respectively.

For the generation of CP, an upstream microchannel inlet was electrically powered, while the side chambers interconnected by a permselective membrane were grounded, configuring the upstream microchannel as the anodic side, as illustrated in Figure 1b,c. The target analytes, having lower diffusion coefficients (e.g., fluorescein molecules and with 0.42 × 10^–9^ m^2^ s^–1^) and lower concentrations in comparison to the background electrolyte ions, were preconcentrated at the edge of the depletion layer in the upstream microchannel from the membrane. This preconcentration resulted from the field-gradient-focusing effect, a consequence of the counteracting interplay between advection (influenced by both pressure-driven and electroosmotic flow) and electromigration, as depicted in Figure 1. The applied voltage and net flow rate for CP ranged from 20 to 30 V and from 50 to 200 nL min^–1^, respectively. We have verified through experiments and numerical simulations (Figure S1) that the net flow is dominated by the forced convection (via the syringe pump, i.e., pressure-driven), with a negligible electroosmotic flow contribution which due to the negatively charged channel walls acts in the same direction as the pressure-driven flow. At the downstream microchannel where the outlet was electrically floating, the depletion layer generated from the membrane propagated toward the end channel mainly by a pressure-driven flow, *u*_0_.

### Decoupled Operation of Periodic CP and Local Electrochemical Sensing

Prior to the application of CP for analyte preconcentration, electrochemical sensing was performed via chronoamperometry and differential pulse voltammetry (DPV) under constant flow and no-flow conditions, respectively (Figure S2). While the DPV signals showed the ability to detect fluorescein concentrations above 70 μM within a diluted PBS (∼0.1 mM) and oxidation event around 0.8 V,^39^ The chronoamperometric signals under constant applied voltage (0.85 V) and flow (50 nL min^−1^) could not distinguish between the different fluorescein concentrations.

**Figure 2:**
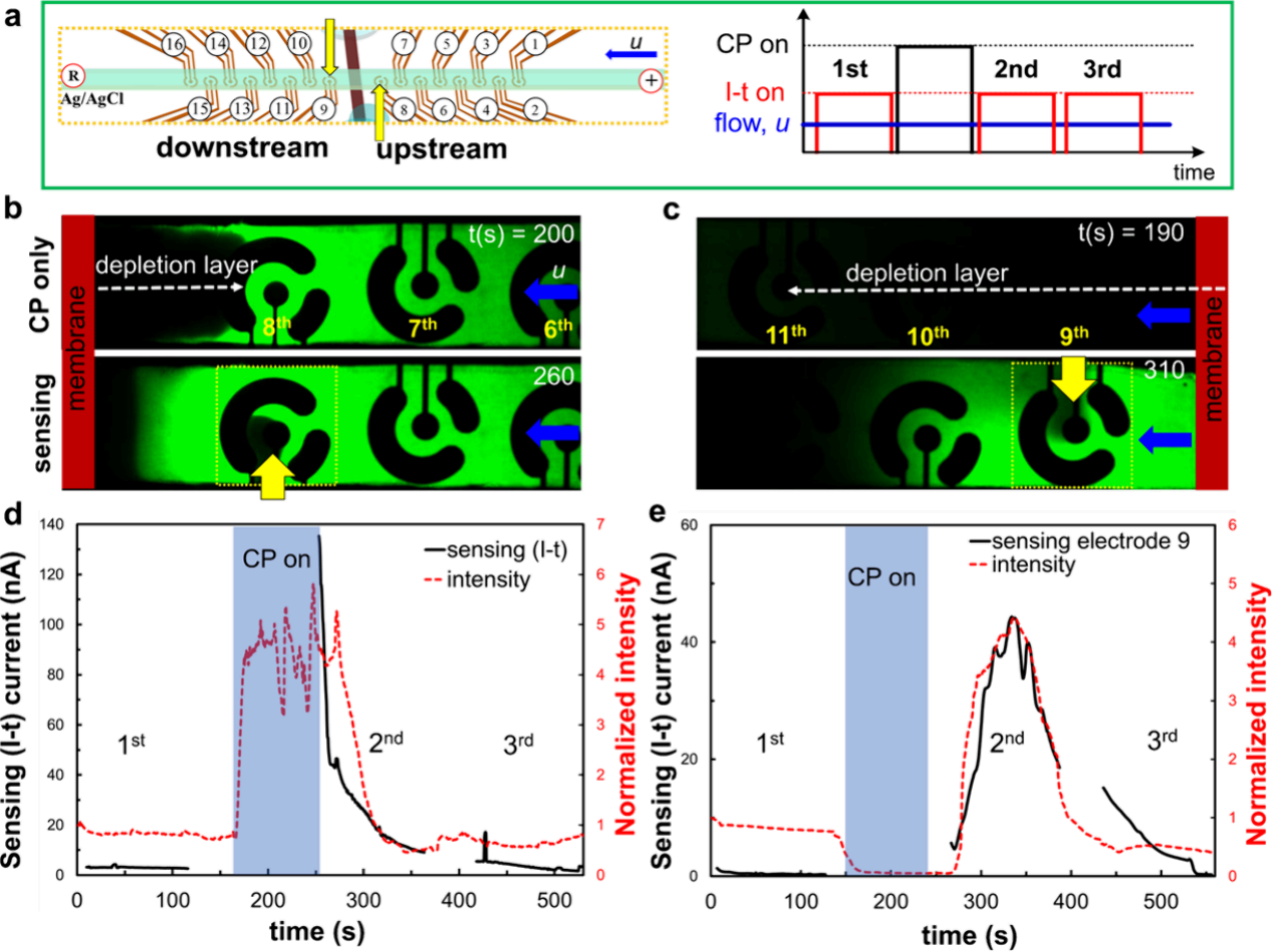
Decoupled CP-based preconcentration of analytes and chronoamperometric (I-t) sensing at the upstream and downstream channel regions. (a) A schematic description showing the sensing locations and time evolving events of CP, local sensing, and constant flow. The target analyte used in the experiments was 150 μM fluorescein in the diluted (0.1 mM) PBS and voltage and flow rate for CP operation were 30 V and 150 nLmin^–1^, respectively. (b) The fluorescence microscopic images representing time evolving event of CP and electrochemical sensing after CP at the (b) upstream (8th electrode) and (c) downstream electrochemical sensors (9th electrode) respectively. The yellow dot-rectangles and yellow arrows indicate the area for averaging fluorescent intensity of fluorescein molecules at the activated electrochemical sensor, respectively. (d, e) The corresponding normalized averaged-sensing area fluorescence intensity profiles (red-dashed line) and chronoamperometric response (black solid line) at the upstream and downstream electrochemical sensors, respectively. The sky-blue rectangles indicate the duration of CP-based preconcentration.

In order to decouple CP operation from local electrochemical sensing, we conducted periodic time-evolved CP events and chronoamperometric sensing under constant flow conditions using local sensors (i.e., 8th and 9th electrodes) positioned in both the upstream and downstream channels from the membrane, as depicted in the schematic setup of Figure 2a. The fluorescence microscopic images in Figure 2b,c (as well as movies S1 and S2) depict representative still shots captured during CP and after CP events in both the upstream and downstream channels, including the activated local sensors. It is clearly seen that the fluorescein molecules were preconcentrated near the eighth electrode positioned at the upstream channel, while the depletion layer propagated downstream along the channel during the CP event. A strong oxidation response, coupled with local depletion were observed above the activated working electrode, coinciding with the release of preconcentrated fluorescein molecules after the cessation of CP (2nd chronoamperometric sensing). The chronoamperometric response at both the eighth and ninth electrodes clearly reveals the effective CP-preconcentration process of the analytes through their corresponding fluorescent intensities and the concurrent increase in electrochemical response following the end of the CP process (Figure 2d,e). Simultaneously, a Gaussian-shape-like signal increase was observed in the downstream channel, primarily resulting from the advection of the preconcentrated plug following its release.

**Figure 3:**
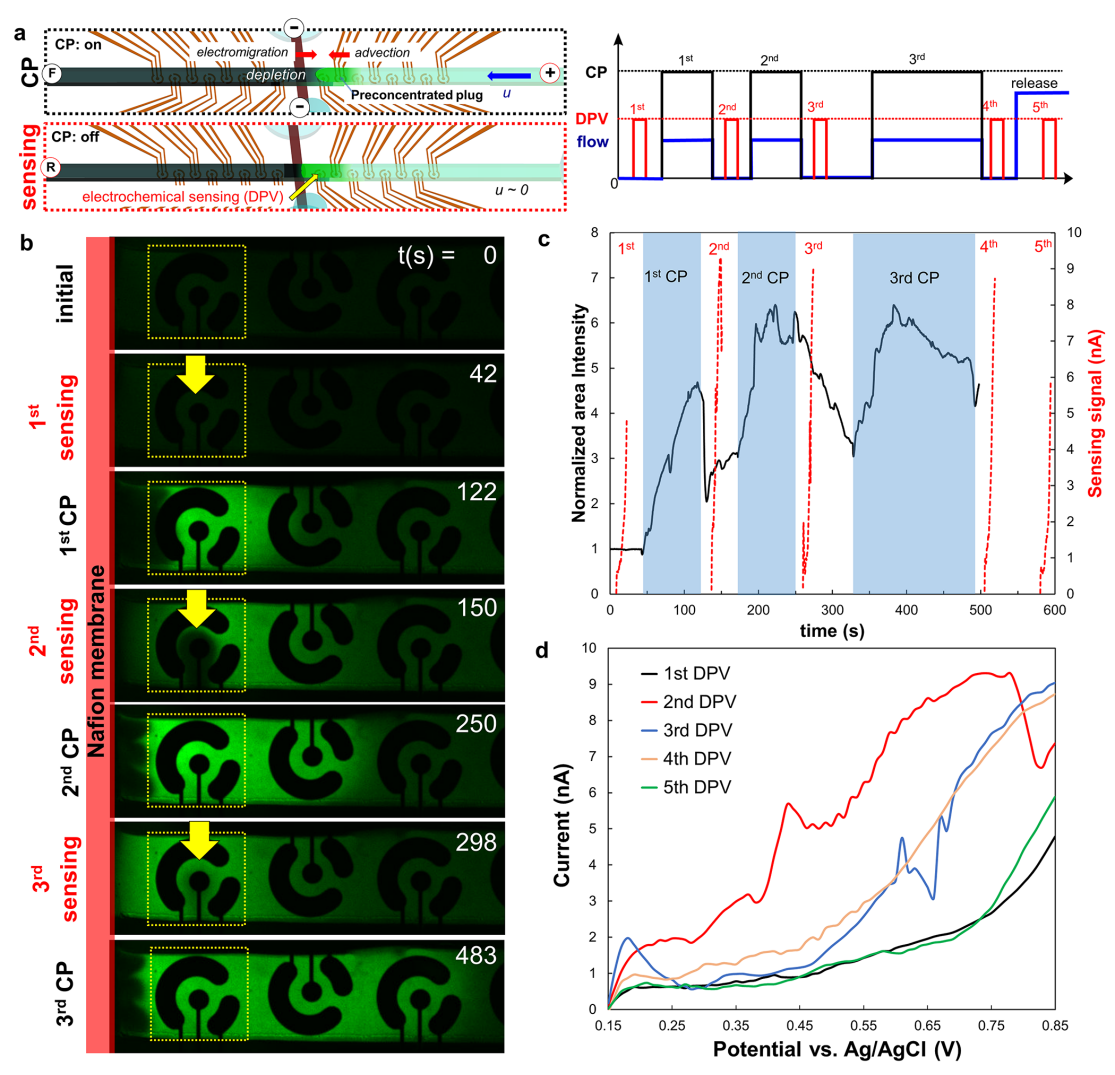
Decoupled CP-based preconcentration of analytes and differential pulse voltammetry (DPV) sensing. (a) Schematic descriptions of time evolving events of periodic CP with flow and DPV sensing at the upstream channel region between CP events without flow application. The used target analyte was 150 μM fluorescein in 0.1 mM PBS and voltage and flow rate for CP operation were 20 V and 50 nL min^–1^, respectively. (b) Fluorescence microscopic images representing the time-evolving event of CP-based preconcentration and electrochemical sensing. The yellow dot-rectangles and yellow arrow indicate the area for averaging fluorescent intensity of fluorescein molecules at the sensor and the event of electrochemical sensing activation, respectively. (c) Corresponding normalized averaged-sensing area fluorescence intensity profiles (black solid line) and sensing (DPV) signals (red dot line) and (d) zoomed-in DPV signals.

In addition, we employed periodic CP applications in conjunction with Differential Pulse Voltammetry (DPV) for electrochemical sensing within the upstream channel region, adopting a decoupled approach from previous chronoamperometric sensing and CP schemes. During the DPV sensing process, fluid flow was halted temporarily and multiple CP events were activated between sensing cycles, as depicted in Figure 3. The results unequivocally revealed a notable increase in fluorescent intensity due to the preconcentrated analytes following the deactivation of CP with minimal advection effects, leading to an enhanced DPV signal (Figure 3b–d and movie S3). When compared with the first sensing cycle before CP application and the fifth sensing cycle after complete release, the results clearly demonstrated the ability to generate multiple CP and sensing events. They provided clear evidence of reproducible analyte preconcentration by CP, manifested through altered fluorescent intensities and the associated enhancement of electrochemical response.

### Continuous Monitoring of Chronoamperometry at the Downstream Channel Region with a Coupled Single CP Operation

**Figure 4:**
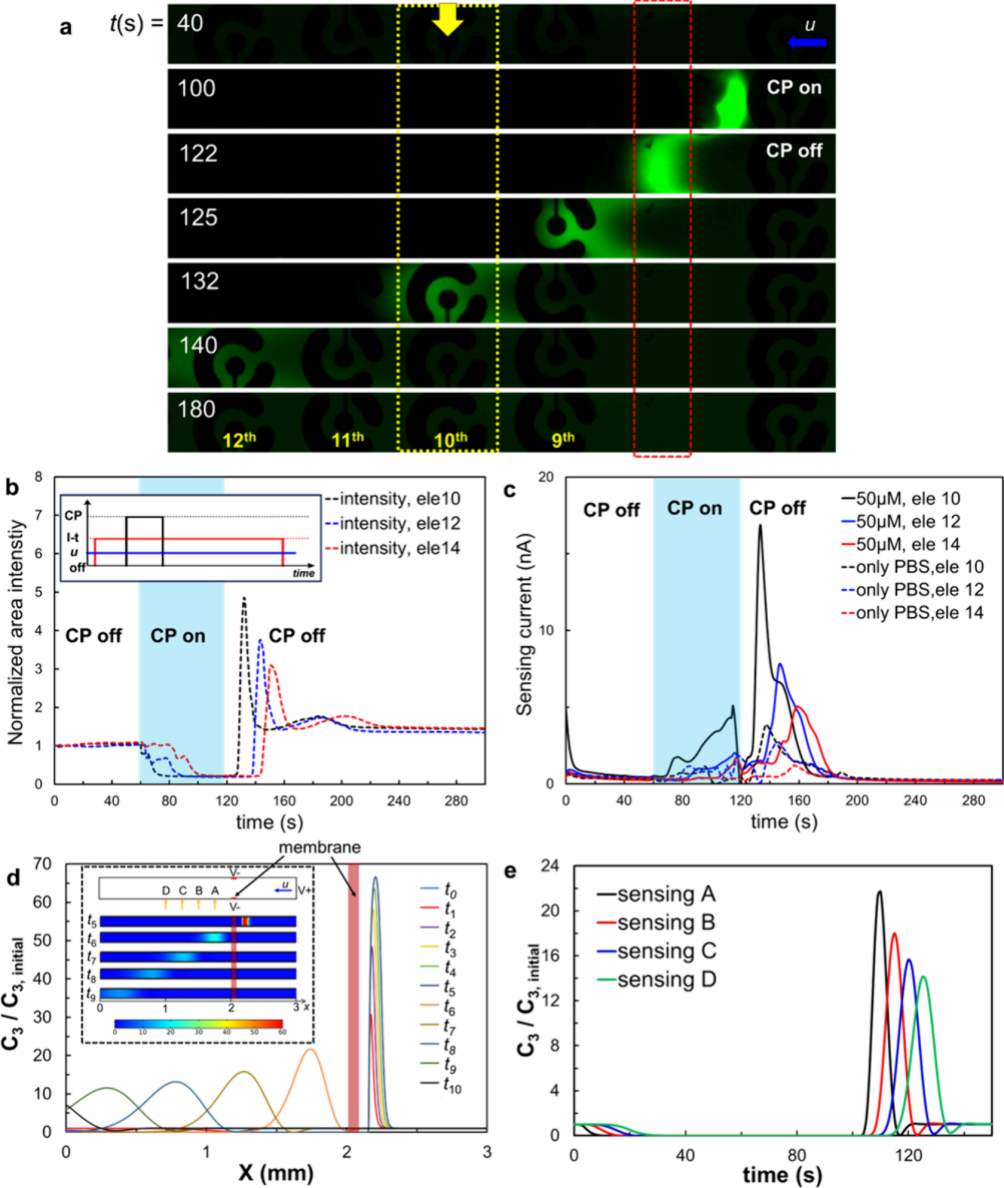
Continuous monitoring of local electrochemical (I-t) sensing at the downstream channel with a coupled CP-based preconcentration. The initial concentration of fluorescein was 50 μM in a diluted PBS. (a) A representative time evolving fluorescence microscopic image at the 10th sensing electrode. (b) The corresponding normalized area-averaged fluorescent intensity of fluorescein molecules and (c) the associated time-lapse chronoamperometric signal at the various sensing electrodes 10, 12, and 14 respectively. See movie S1 for the time-evolving CP and electrochemical sensing event at the various electrodes. (d) Numerical simulation of time evolving preconcentration of molecules (*C*_3_) under the CP (*t*_0_ to *t*_5_) and downstream advection of preconcentrated *C*_3_ by deactivating CP under advection, *u* (*t*_6_–*t*_10_). The inset supports (a). (e) Numerical simulation of time-lapse normalized *C*_3_ at the various sensor locations downstream of the membrane, taken from the inset of (d).

In the coupled CP and electrochemical sensing setup for continuous monitoring throughout the entire process, various sensors (i.e., 10th, 12th, and 14th) positioned at the downstream channel were employed with the chronoamperometric method in separate experiments under single CP operating conditions having 20 V and 100 nL min^–1^ in voltage and flow rate, respectively. Figure 4a shows the fluorescence of the analyte (50 μM fluorescein within 0.1 mM PBS) throughout the entire CP and sensing cycle (movie S4). The area-averaged fluorescent intensity at the different sensing areas (Figure 4b) of the corresponding sensing electrodes, reflecting the concentration of fluorescein, closely parallels the simultaneously obtained electrochemical sensing signals (Figure 4c; see also, Figure S3). Also, it can be clearly seen that as the sensors are located further downstream (from 10th to 14th electrode), the chronoamperometric signal peak shifts to a later time while diminishing in amplitude, primarily due to the diffusion and advection of the analyte downstream under constant flow. It is noteworthy that the increase in sensing signal during CP, despite the complete depletion occurring, may be due to cross-talk between the CP driven applied electric field and that used for the electrochemical sensing. Additionally, when we conducted coupling of CP and electrochemical sensing in the upstream microchannel, electrolysis and vigorous bubble formation were observed at the active sensors, resulting from the cross-talk between the external electric fields for inducing CP to those used for measuring the electrochemical reactions (movie S5). Two-dimensional fully coupled transient simulations (Figure 4d,e), employing similar system parameters for CP and sensing time events, qualitatively validated the complete depletion at the downstream channel during CP, as well as the serial increase in the fluorescence signal along the location of the sensor following the release event after CP. Furthermore, these simulations confirmed the decrease in amplitude and the shift to a later time in the concentration of the target analyte, closely mirroring the electrochemical sensing measurements.

**Figure 5:**
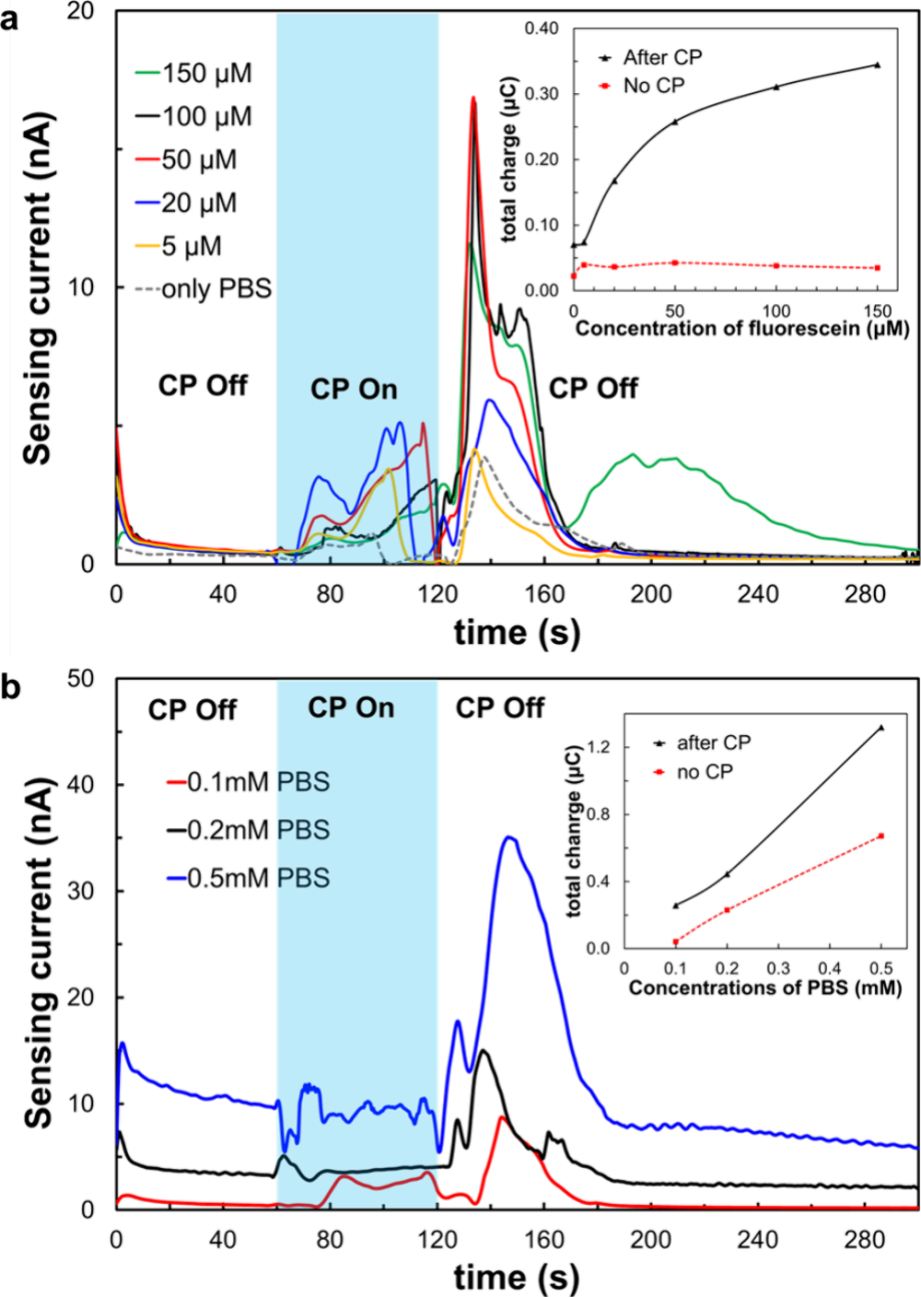
Efficiency of preconcentration of molecules in electrochemical sensing at the downstream channel region for (a) various fluorescein concentrations in a 0.1 mM PBS using the 10th electrode and (b) various electrolyte concentrations with a fixed 50 μM fluorescein concentration using the 12th electrode. The inset graphs in parts a and b are the total charge as a function of the various concentrations of fluorescein and PBS, respectively.

For a comprehensive quantitative analysis, we applied various concentrations of fluorescein molecules using the same coupling method and CP parameters, as shown in Figure 4 (Figure 5a). As anticipated, the peak of sensing signals increased with increased analyte concentrations. Notably, even in a pure PBS solution, a distinct peak in the electrochemical sensing current was observed, indicating the impact of CP on the bare PBS solution. This phenomenon is likely attributed to rapid changes in the background ion concentration near the edge of the depletion layer induced by CP, variations in ion contents within PBS (e.g., HPO_4_^–2^ and H_2_PO_4_^–1^ with lower diffusion coefficients),^40^ or pH fluctuations.^41^ Further study is required to better understand this phenomenon. We further quantified the total charges (see inset of Figure 5a) during the time periods with no CP application and following the CP by integrating the currents over specific time intervals (0 to 60 s and 120 to 180 s, respectively). The results revealed that the sensing signals exhibited a more pronounced increase as a function of analyte concentration in the case of with CP, while no significant signal difference was observed without CP. Additionally, we conducted experiments with various concentrations of electrolyte while keeping the fluorescein concentration fixed at 50 μM (Figure 5b). In general, the signals displayed a positive correlation with the concentration of the background electrolyte. Moreover, significant signal peaks were observed at all applied concentrations after CP, beyond the 120 s mark, in response to the release of preconcentrated fluorescein at all concentrations. When comparing the total charge, the signals exhibited an almost linear increase with increased PBS concentrations in both CP and no CP cases, where the former exhibited a larger signal. These results highlight the real-time monitoring capability of electrochemical sensing signals within the downstream channel during the entire CP operation.

### Enhancement of Homovanillic Acid (HVA) Detection in the Downstream Channel with a Coupled CP Operation

Furthermore, we endeavored to detect homovanillic acid (HVA), an important catecholamine metabolite in the central nervous system.^42,43^ Prior to sensing of the HVA target analyte via chronoamperometry with continuous monitoring with CP-based preconcentration, we systematically conducted electrochemical sensing across varying concentrations of HVA in a fixed dilute PBS concentration (σ = 0.23 mS cm^–1^; Figure S4). This was executed within a macrochamber with relatively large-sized embedded rectangular gold working/counter electrodes, alongside our microfluidic system (insets in Figure S4). DPV signals from both macro- and microfluidic setups unveiled an oxidation peak of HVAs at approximately 0.7 V. The limit of detection (LOD) for HVA spanned from 0.05 to 0.1 and 0.1 to 0.25 mM for the macro- and microfluidic setups, respectively. Analogous LOD values were obtained from chronopotentiometry signals under a sustained applied voltage of 0.7 V by using our electrochemical sensor in the microfluidic system. Optimized parameters for CP-based preconcentration were 32 V and 100 nL min^–1^ obtained from using 2.5 μM fluorescein molecules as a model for the invisible target analyte (HVA). We have used the coupled electrochemical sensing at the downstream sensors and single CP operation due to the impractical challenges associated with the decoupled mode in the upstream channel, specifically, the lack of continuous monitoring as the upstream sensors cannot operate simultaneously to the CP as well as the inability to precisely predicting the location of the invisible target analyte’s preconcentrated plug.

**Figure 6:**
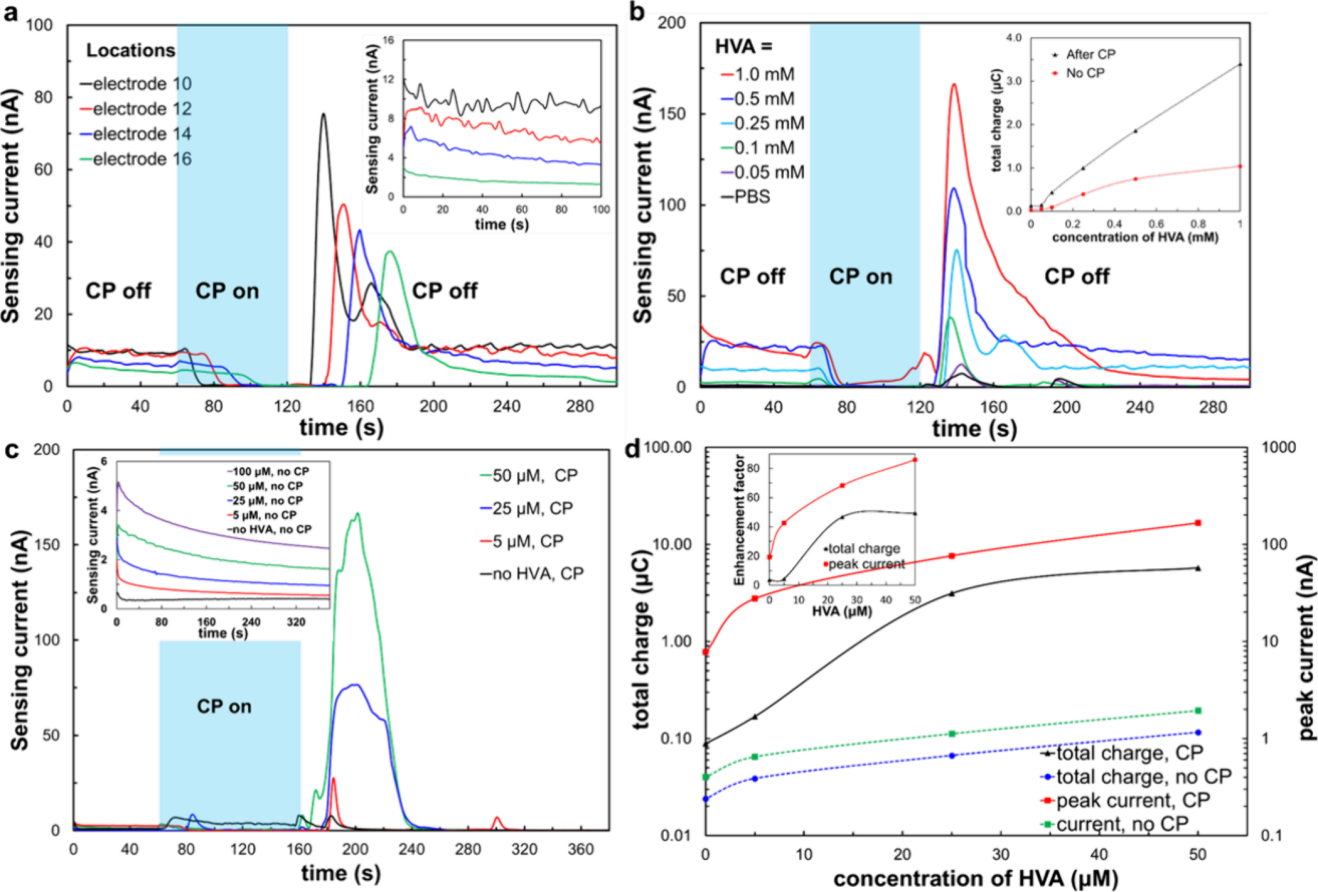
Enhancement of the HVA detection signal using chronoamperometric sensing coupled with single CP-based preconcentration. (a) Time-lapse chronoamperometric signal at the various sensing electrodes (10th, 12th, 14th, and 16th electrode) using a fixed 0.25 mM HVA in diluted PBS. The sky-blue rectangle regime indicated the application of a CP. The insets show chronoamperometric signals of HVA under no CP conditions at various sensing locations while electrode lines for CP were still connected to the Keithley power source. (b) Time-lapse chronoamperometric signal at the 10th electrode for varying HVA concentrations in diluted PBS. The inset graph is the total charge (i.e., time integral of the current) as a function of the various concentrations of HVA. (c) Time-lapse chronoamperometric signal at the downstream electrode (10th) for varying HVA concentrations in diluted artificial sweat (0.75 mS cm^–1^). The sky-blue rectangle regime indicated the application of CP with 100 s duration. The inset graph depicts the sensing signals without CP. (d) Total charge (i.e., time integral of the current) and peak current as a function of the various concentrations of HVA in diluted artificial sweat. The inset graph depicts the enhancement factor by CP in comparison to sensing signals without CP along varying HVA concentrations.

In Figure 6, chronoamperometry signals unveil sensor responses at various downstream locations (10th, 12th, 14th, and 16th electrodes), each exposed to a fixed 0.25 mM HVA concentration, and different HVA concentrations sensed at the 10th electrode. Employing continuous monitoring and single CP preconcentration, both configurations exhibit a pronounced HVA sensing peak following CP application. Notably, at different sensing locations (Figure 6a), the chronoamperometric signal peaks systematically shift to a later time as the sensors are positioned further downstream, aligning with the observed trend in Figure 4. Intriguingly, a substantial decrease in the value of the sensing signals occurred during the CP activation at different times, corresponding to the downstream propagation of the depletion layer to the different locations of the sensors (Figure 6a). This phenomenon may be attributed to diminished cross-talk, between the electrical sensing and that of the applied CP, owing to the lower applied voltage (0.7 V) utilized for electrochemical sensing, in contrast to that used before, as detailed in Figures 4 and 5. The sensing signals of HVA (0.25 mM) under no CP conditions decreased as the distance from the reference electrode decreased (inset of Figure 6a). While atypical, this trend may be associated with crosstalk to CP grounding near the membrane close to electrode 10, necessitating further meticulous investigation. Examination of diverse HVA concentrations revealed a distinct monotonic increase in the peak of sensing signals with increasing analyte concentrations (Figure 6b). Notably, the total charges exhibited a more pronounced increase proportional to the analyte concentration in the CP case compared with the no CP case (inset of Figure 6b).

To further enhance the practical implications of our approach, we conducted additional experiments employing an artificial sweat solution (1:10 dilution, 0.75 mS cm^–1^) with a HVA concentration range from 5 to 50 μM (Figure 6c,d) that is relevant for practical sensing applications of HVA.^44,45^ Due to the higher conductivity of the background electrolyte, the duration of CP with operation parameters (45 V, 100 nL min^–1^) was extended from 60 to 100 s to ensure the generation of a sufficiently preconcentrated analyte plug at the upstream channel. Analogous to the findings in Figure 6b, a clear increase in the sensing signal was obtained for increased analyte concentrations without CP (inset of Figure 6c). Furthermore, both the total charges and peak currents exhibited a significant enhancement (up to 80) that is proportional to the analyte concentration with CP versus the case of no CP. These results, illustrating significant enhancement of the detection sensitivity of HVA upon electrokinetic preconcentration, highlight the system’s potential and versatility for sensitive detection of a diverse range of biomolecules.

## Conclusions

In summary, we integrated two technologies, concentration polarization (CP)-based preconcentration of analytes and local electrochemical sensing, realized via an array of sensors within a microfluidic system to significantly enhance the detection signal. Both decoupled and coupled CP with local electrochemical sensing strategies at either upstream and downstream channel regions relative to the membrane have revealed significant enhancements in signal detection through the application of chronoamperometry and differential pulse voltammetry techniques. In particular, at the coupled configuration we have successfully obtained continuous monitoring of the chronoamperometric response within the downstream channel with periodic CP application and validated their performance using various positions of electrochemical sensors, a range of target analyte, and electrolyte ionic concentrations, thereby demonstrating its ability to detect charged target analytes with improved signal sensitivity and limit-of detection. As a practical application, we have demonstrated the significantly enhanced (10–100 enhancement factor) detection sensitivity of relevant HVA concentrations using combined CP-based preconcentration with coupled electrochemical sensing. However, we also identified certain limitations when our system is configured in coupled mode. First, coupling CP and electrochemical sensing in the upstream microchannel proved unfeasible as the concurrent operation led to vigorous electrolysis and bubble formation, causing electrode degradation. Furthermore, challenges arose when using high-conductivity solutions, necessitating higher CP voltages, which in turn enhanced the risk of electrolysis within the depleted solution areas. In addition, the preconcentration of charged ionic species led to unexpected fluctuations in background electrolyte concentrations, consequently affecting signal enhancements that cannot be directly attributed to the target analyte. In future research endeavors, we plan to design an innovative microfluidic setup, e.g., featuring a Y-channel configuration, to accommodate multiple target samples with varying ionic charges, including neutral and negatively charged species. Additionally, our focus will be on further enhancing sensor performance, with particular emphasis on selectivity while suppressing potentially inhibiting molecules.

## Experimental Section

### Microfluidic Chip Combining CP and Local Electrochemical Sensing

To fabricate the microfluidic chip (Figure 1), an array of electrochemical sensing electrodes was patterned onto a 1 mm thick glass slide using a standard photolithography for lift-off processes. Then, layers of Au/Cr (200 nm/30 nm in thickness) were deposited and lifted off onto the patterned glass slide. The design of the electrochemical sensing array was based on the previous work.^46^ A Nafion membrane was fabricated onto the electrode-patterned glass slide using microchannel flow-based patterning method, as previously described.^47,48^ To create the thin spacer with a microchannel 500 μm in width and 18 mm in length, 50 μm thick double-sided tape (3M) was cut using an electronic cutting machine (Silhouette Cameo 3, Silhouette America Inc.). For the top cover plate, we used a polydimethylsiloxane (PDMS) block, including inlet and outlet holes (2 mm in diameter) of the main channel and side chambers (2 mm in diameter). In addition, large PDMS reservoirs with 8 mm and 3.5 mm in diameter were stacked above inlet/outlet holes and side chamber respectively using O_2_ plasma treatment to avoid evaporation and to connect the external reference electrode. All of these components were manually assembled by sandwiching the bottom glass slides and top PDMS block. This configuration is integral to the functionality of the microfluidic system, allowing controlled fluid flow and electrochemical sensing and thus facilitating the investigation of various chemical and electrochemical processes. The detailed design and operation of CP were adopted from our prior works.^47,48^ Each sensing electrode within the system possesses the capability to establish a three-electrode sensing setup, comprising a reference electrode, a working electrode, and a counter electrode, on demand. However, for simplicity, we employed a common external reference electrode placed in the outlet reservoir. The membrane was located in the middle of the electrode array in the main channel and was interconnected to the side chambers.

### Experimental Setup for CP and Local Electrochemical Sensing

For CP generation, an external electrode (1 mm diameter) was inserted at the inlet of the main channel functioning as the anode electrode, while two patterned gold electrodes were integrated into side chambers as cathode electrodes, respectively. This electrode configuration was connected to a voltage source (Keithley 2636) to establish a CP with a current pathway restricted to the upstream channel as depicted in Figure 1b. The inlet flow, ranging from 50 to 200 nL min^–1^, was meticulously controlled by using a syringe pump (KDS legato 210, KDScientific). To ensure the chip’s optimal performance and cleanliness before conducting experiments, we followed a comprehensive wetting and cleaning protocol which were previously described elsewhere.^49,50^ For visualization of the CP-based preconcentration, we employed negatively charged fluorescein molecules (Sigma). All experiments were recorded by using a spinning disc confocal microscopy system (Yokogawa CSU-X1) connected to an inverted microscope (Eclipse Ti–U, Nikon) and a camera (Andor iXon3). Their fluorescent intensity as a function of time was further analyzed by normalizing the average area intensity by the initial value, utilizing a Matlab program for data analysis.

All electrochemical measurements were performed using a PalmSens4 (Palmsens, Ltd.) and in a three-electrode cell configuration consisting of the microfabricated electrodes (working electrode, “W”, and counter electrode, “C”) and an externally applied commercial Ag/AgCl electrode (CHI111P, CH Instruments, reference electrode, “R”) allocated in the outlet of the microchannel. All electrochemical potential values are versus the Ag/AgCl half-cell potential.

### Numerical Simulation

The Poisson–Nernst–Planck–Stokes (PNPS) equations were solved using a fully coupled, two-dimensional (2D) time-dependent model (see the Supporting Information for more details on the numerical simulations) using COMSOL Multiphysics 5.3. Due to computational limitations in covering the broad spectrum of length scales that exist in the problem and since the numerical simulations were intended only for qualitative comparison with the experiments, a nonrealistic large Debye length was used.

## Supporting Information Available

Detailed numerical simulations methodology; Supplementary figures showing local electrochemical sensing under various concentrations of fluorescein molecules in a diluted PBS; Continuous monitoring of local electrochemical (I-t) sensing at the downstream channel with a CP-based preconcentration; Local electrochemical sensing under various concentrations of HVA in a diluted PBS (PDF)
Movie S1: Decoupled CP-based preconcentration of analytes and chronoamperometric sensing at the upstream channel region (MP4)
Movie S2: Decoupled CP-based preconcentration of analytes and chronoamperometric sensing at the downstream channel region (MP4)
Movie S3: Decoupled CP-based preconcentration of analytes and DPV sensing at the upstream channel region (MP4)
Movie S4: Continuous monitoring of local electrochemical (I-t) sensing at the downstream channel region with CP-based preconcentration (MP4)
Movie S5: A vigorous electrolysis and bubble formation during the coupled operation of CP and electrochemical sensing in the upstream microchannel (MP4)
