# Supplementary material for: On-Chip Electrochemical Sensing with an Enhanced Detecting Signal Due to Concentration Polarization-Based Analyte Preconcentration
Source: Anal Chem. 2024 Apr 9;96(16):6501–10. doi: 10.1021/acs.analchem.4c01018 (PMC11044107; doi:10.1021/acs.analchem.4c01018)
Supplement: Supplementary file 1 — ac4c01018_si_001.pdf [file ac4c01018_si_001.pdf]

Supporting information

On-chip electrochemical sensing with enhanced detecting signal due to concentration-polarization based analyte preconcentration

Sinwook Park[1, 3], Daniel Kaufman[2], Hadar Ben-Yoav[2], and Gilad Yossifon[1, 3]*

[1]School of Mechanical Engineering, Tel-Aviv University, Tel Aviv, 6997801, Israel

[2]Nanobioelectronics Laboratory (NBEL), Department of Biomedical Engineering, Ben-Gurion University of the Negev, Beer-Sheva, 8410501, Israel

[3]Department of Biomedical Engineering, Tel-Aviv University, Tel Aviv, 6997801, Israel

* ***Corresponding Author***

E-mail: gyossifon@tauex.tau.ac.il

Numerical Simulations Methodology

Here, we account for the preconcentrated fluorescein molecules as a third ionic species, C_3, at upstream channel from the membrane under the CP and downstream advection of preconcentrated C_3 by deactivating CP under advection within a microchannel-membrane system, via a fully coupled, two-dimensional (2D), time-dependent model, using the finite-element based software COMSOL (V5.3). The governing equations in the system are the continuity of ions, C_i, expressed using the Nernst−Planck relation for the ion fluxes, j_i

$$\frac{\partial c_i}{\partial t} = -\nabla \cdot j_i, \quad j_i = -\left(D_i \nabla c_i + Z_i \frac{F}{RT} D_i c_i \nabla \phi \right) + u c_i, \tag{S1}$$

the Poisson equation for the electric potential, ϕ, in terms of the excess ionic charge density, ρ_e

$$-\nabla \cdot \left(\varepsilon \nabla \phi \right) = \rho_e; \quad \rho_e = F \sum_i z_i c_i, \tag{S2}$$

the incompressible Stokes equations

$$\rho \frac{\partial u}{\partial t} = -\nabla p + \eta \nabla \cdot \nabla u - \rho_e \nabla \phi, \qquad \nabla \cdot u = 0\,. \tag{S3}$$

Herein, F is the Faraday constant, R is the universal gas constant, T is the temperature, p is the pressure, ρ is the fluid mass density, η is dynamic viscosity, and ε is permittivity of solution at room temperature. We use i_1 for K^+, i_2 for Cl^- and i_3 for negatively charged fluorescein molecules. z_i and D_i are the valence and diffusion coefficient of species i, respectively, where $D_1 = 2.0\times10^{-9} m^2 s^{-1}$, $D_2 = 2.0\times10^{-9} m^2 s^{-1}$, $D_3 = 4.2\times10^{-10} m^2 s^{-1}$, $z_1 = -z_2 = -z_3 = 1$. The values of D_3 and z_3 were chosen from the properties of fluorescein[1].

The numerical model of the system, representing the cross-sectional side view, consisted of a cation-permselective membrane (length l = 80 μm) located at the center of the bottom surface of a straight, long microchannel (length L = 4 mm, height H = 40 μm mm) (inset in Fig. 4d). The initial concentrations of ions $c_{i,initial}$ were $c_{1,initial}$, $c_{2,initial} = 1\times10^{-5}$, $c_{3,initial} = 5\times10^{-8} mol\cdot m^3$ respectively. The boundary conditions at the membrane interface ($x\in[-0.5l, 0.5l]$) were no penetration of anions ($j_- = 0$, i.e. ideal cation-permselective membrane), fixed cation concentration ($c_1 = N\cdot c_{1,initial}$), and Donnan potential ($-RT/F\cdot\ln(N)$), with a value of N = 5. At the microchannel inlet ($x = -0.5L, y\in[0,H]$), a constant bulk concentration ($c_1 = c_{1,initial}$, $c_2 = c_{2,initial}$, $c_3 = c_{3,initial}$) was imposed along with a net flow (i.e., uniform inlet velocity u) and the electric potentials ($V = 40\,\mathrm{V}$) were applied. Electro-osmotic flow (EOF) was accounted for by using the Helmholtz-Smoluchowski slip velocity with typical zeta potential of −25 mV at the channel's walls. At the microchannel outlet ($x = 0.5L, y\in[0,H]$), open boundary conditions ($\nabla c_i \cdot n = 0,\ du/dx = 0$) and floating potential ($\mathrm{D}\cdot\mathrm{n} = 0$, where D is displacement of electric field) were imposed. At the other channel walls, no-penetration, electrical insulation, and no-slip conditions were used for the hydrodynamic problem.

Supporting figures

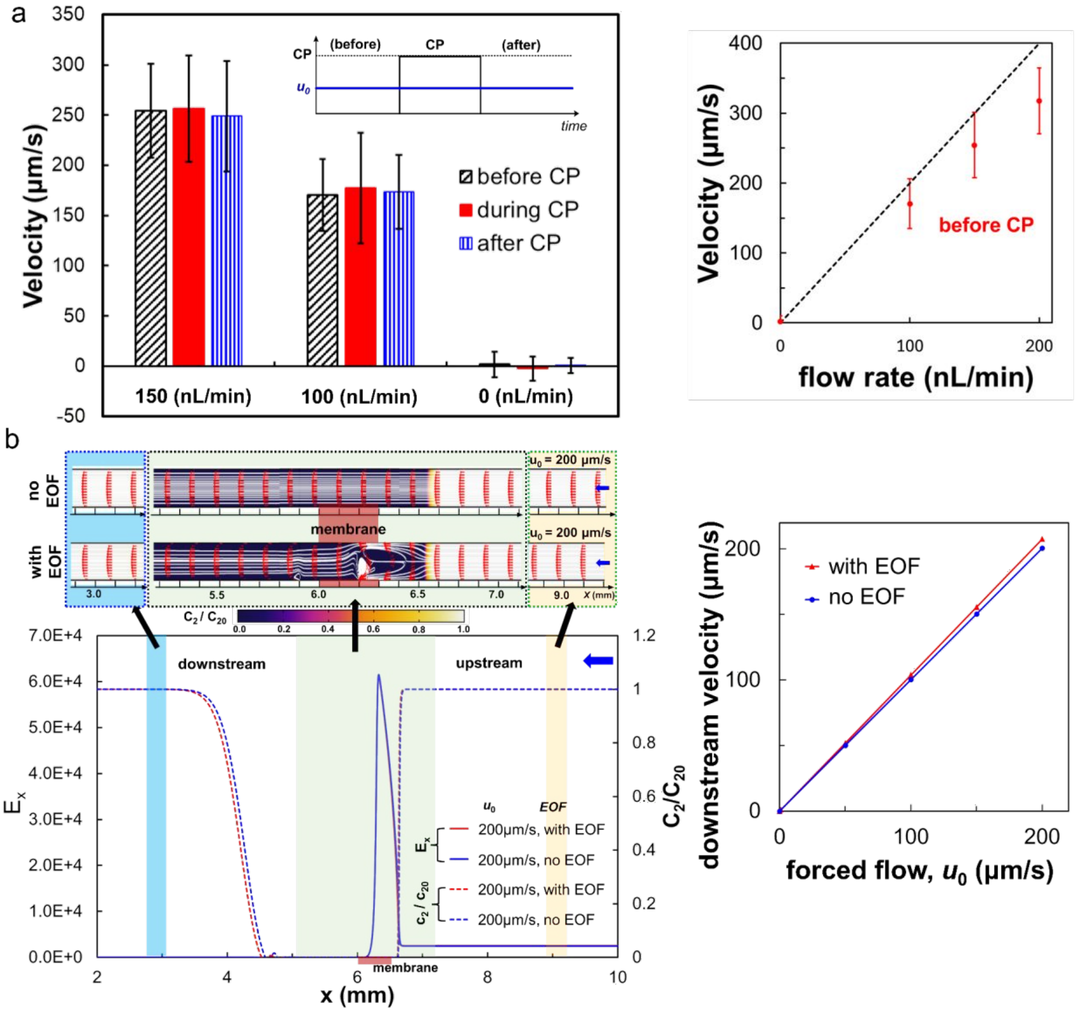

Figure S1. The role of forced flow (u_0) and electroosmotic flow (EOF) during CP operation. (a) Experimentally measured cross-sectional averaged velocities in the downstream channel region (3.5mm away from the membrane interface) with/without CP operation under different forced-flow rates (u_0 = 0, 100, and 150 nL min^{-1}) applied by a syringe pump. On the right – the measured cross-sectional averaged velocity solely driven by the syringe pump, shows <20% deviation from the theoretical prediction (black dashed line). (b) Representative 2D simulation results illustrating the effect of EOF under a forced-flow velocity of u_0 = 200 μm s^{-1}. The normalized cation concentrations (c_2) indicates ion depletion as expected. White lines and red arrows indicate 2D velocity streamlines and vector fields, respectively. The blue arrow indicates the direction of u_0. On the right - the cross-sectional averaged velocity at the downstream channel (x = 3 mm) versus varying forced-flow velocities u_0 with/without EOF effect.

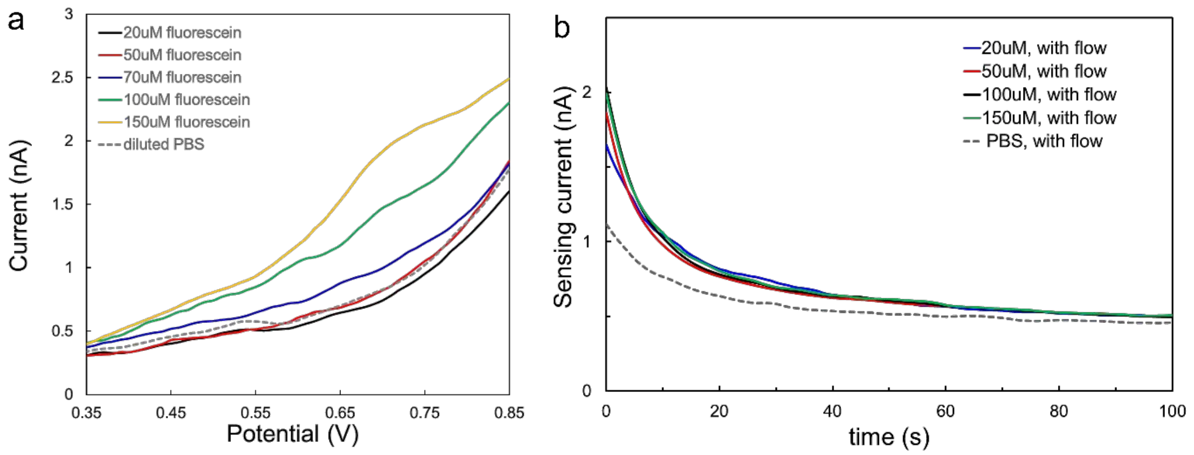

Figure S2. Local electrochemical sensing under various concentrations of fluorescein molecules in a diluted PBS. (a) Differential pulse voltammetry without flow. (b) Chronoamperometric response under application of constant voltage of 0.85V with flow of 50 nL min^{-1}.

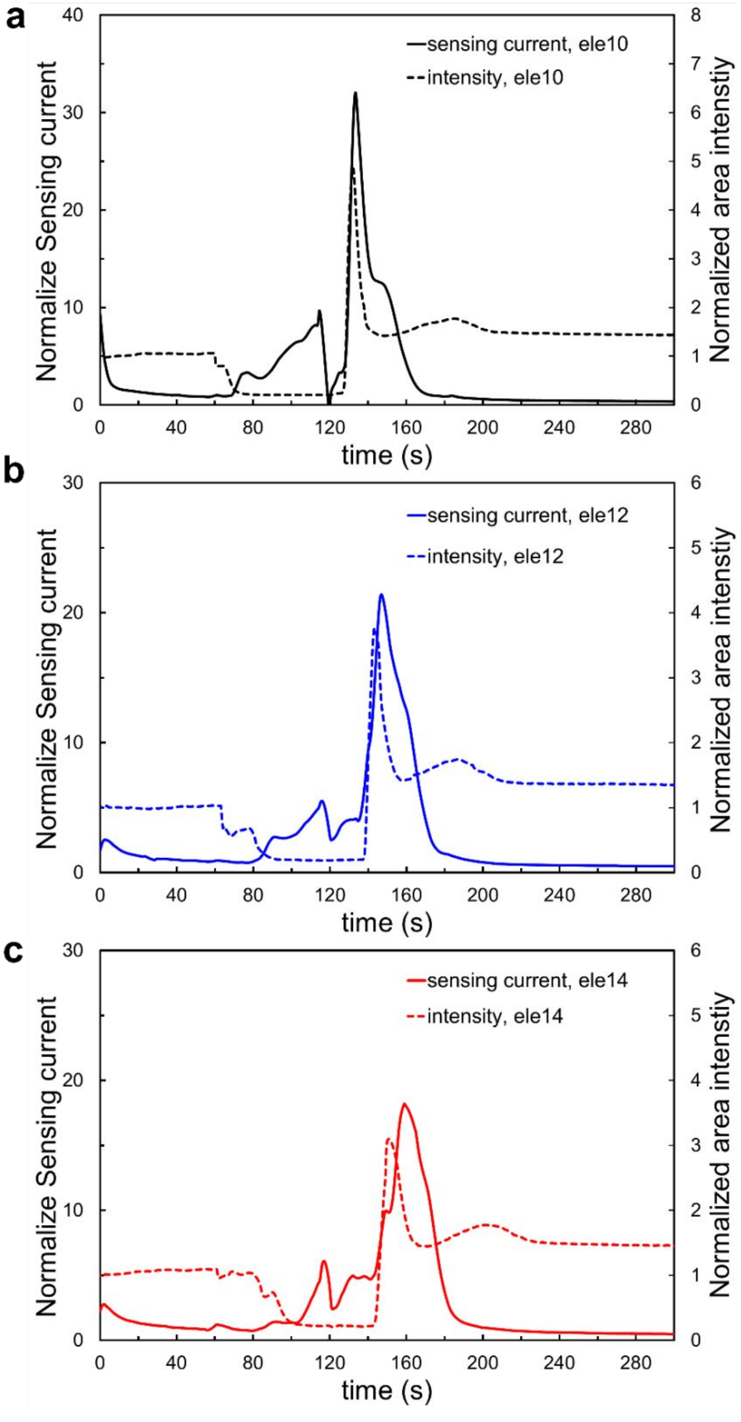

Figure. S3. Continuous monitoring of local electrochemical (I-t) sensing at the downstream channel with a CP-based preconcentration. The time-transient sensing signal synchronized with the normalized fluorescent intensity at each sensing location of (a) 10th electrode, (b) 12th electrode and (c) 14th electrode, taken from figure 4b and c.

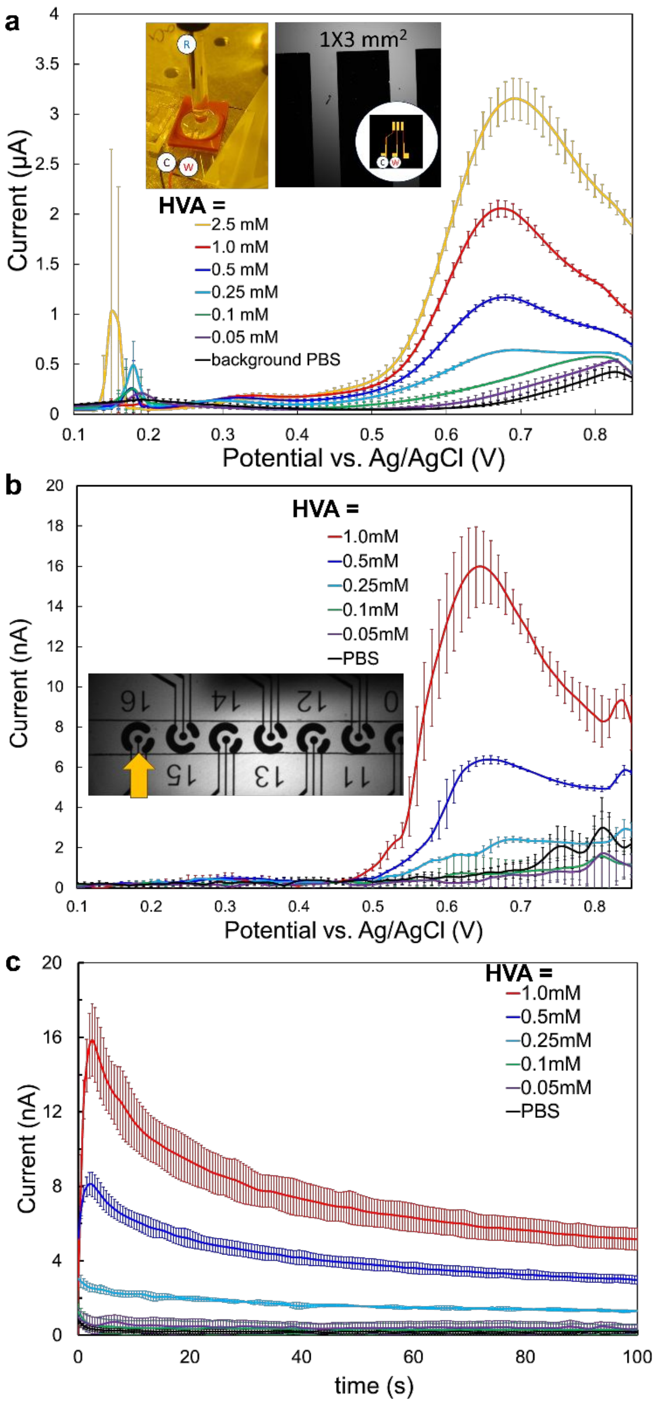

Figure. S4. Local electrochemical sensing under various concentrations of HVA in a diluted PBS. (a) Differential pulse voltammetry using a macro-chamber with relatively large-sized embedded rectangular gold working/counter electrodes and (b) a microfluidic system (16th electrode) without flow. (c) Chronoamperometric response under application of constant voltage of 0.7V with flow of 100 nL min^{-1} at 16th electrode in the microfluidic chip.

Supporting movies

Movie S1: Decoupled CP-based preconcentration of analytes and chronoamperometric sensing at the upstream channel region. (top) time-evolution of the preconcentration event of fluorescein molecules and their transport to downstream locations following deactivation of CP corresponding to Fig. 2b. (bottom) The transient normalized averaged-sensing area fluorescence intensity profiles (red-dashed line) and chronoamperometric response (black solid line) corresponding to Fig. 2b.

Movie S2: Decoupled CP-based preconcentration of analytes and chronoamperometric sensing at the downstream channel region. (top) time-evolution of the preconcentration event of fluorescein molecules and transport to downstream locations after deactivation of CP corresponding to Fig. 2c. (bottom) The transient normalized averaged-sensing area fluorescence intensity profiles (red-dashed line) and chronoamperometric response (black solid line) corresponding to Fig. 2e.

Movie S3: Decoupled CP-based preconcentration of analytes and DPV sensing at the upstream channel region. Time-evolution of the preconcentration event of fluorescein molecules through multiple CP operation and DPV sensing.

Movie S4: Continuous monitoring of local electrochemical (I-t) sensing at the downstream channel region with CP-based preconcentration. (top) three recorded time-evolution of the preconcentration event of fluorescein molecules and transport to downstream locations following deactivation of CP with various sensing locations (bottom left) the normalized area-averaged fluorescent intensity of target analyte and (bottom right) continuous chronoamperometric response of various sensors corresponding to Fig. 4b and c, respectively.

Movie S5: A vigorous electrolysis and bubble formation during the coupled operation of CP and electrochemical sensing in the upstream microchannel.

References

(1) Casalini, T.; Salvalaglio, M.; Perale, G.; Masi, M.; Cavallotti, C. Diffusion and Aggregation of Sodium Fluorescein in Aqueous Solutions. J. Phys. Chem. B 2011, 115 (44), 12896–12904.
